# Synergistic inhibition of tumor growth by combination treatment with drugs against different subpopulations of glioblastoma cells

**DOI:** 10.1186/s12885-017-3924-y

**Published:** 2017-12-29

**Authors:** Chia-Hsin Chang, Wei-Ting Liu, Hui-Chi Hung, Chia-Yu Gean, Hong-Ming Tsai, Chun-Lin Su, Po-Wu Gean

**Affiliations:** 1 Department of Pharmacology, College of Medicine, National Cheng Kung University, Tainan, Taiwan; 2 Department of Diagnostic Radiology, National Cheng Kung University Hospital, Tainan, Taiwan; 3 Department of Biotechnology and Bioindustry Sciences, College of Bioscience and Biotechnology, National Cheng Kung University, Tainan, Taiwan

**Keywords:** Glioma, Cancer stem cells, Minocycline, STAT3, Combination therapy, Synergy

## Abstract

**Background:**

Glioma stem cells (GSCs) contribute to tumor recurrence and drug resistance. This study characterizes the tumorigenesis of CD133^+^ cells and their sensitivity to pharmacological inhibition.

**Methods:**

GSCs from human U87 and rat C6 glioblastoma cell lines were isolated via magnetic cell sorting using CD133 as a cancer stem cell marker. Cell proliferation was determined using the WST-1 assay. An intracranial mouse model and bioluminescence imaging were used to assess the effects of drugs on tumor growth in vivo.

**Results:**

CD133^+^ cells expressed stem cell markers and exhibited self-renewal and enhanced tumor formation. Minocycline (Mino) was more effective in reducing the survival rate of CD133^+^ cells, whereas CD133^−^ cells were more sensitive to inhibition by the signal transducer and activator of transcription 3 (STAT3) inhibitor. Inhibition of STAT3 decreased the expression of CD133^+^ stem cell markers. The combination of Mino and STAT3 inhibitor synergistically reduced the cell viability of glioma cells. Furthermore, this combination synergistically suppressed tumor growth in nude mice.

**Conclusion:**

The results suggest that concurrent targeting of different subpopulations of glioblastoma cells may be an effective therapeutic strategy for patients with malignant glioma.

## Background

Glioblastoma multiforme (GBM) is the most common type of primary brain tumor. Its infiltrative nature prevents complete resection [1]. In addition, GBM is highly resistant to radiation and chemotherapy. The median survival time is around 1–2 years [2]. Therefore, it is imperative to develop novel strategies and to identify more efficient therapeutic approaches for the treatment of GBM.

Accumulating evidence indicates that a small populaton of cells within the malignant neoplasm are capable of initiating and promoting tumor growth [3, 4]. These cells, termed cancer stem cells (CSCs) or tumor-initiating cells (TICs), can form neurospheres in vitro and initiate tumor growth in nude mice [3]. Thus, CSCs are thought to contribute to tumor recurrence and drug resistance after conventional treatment [5].

CSCs were first isolated from tumor tissues and later from tumor cell lines, including breast cancer, prostate cancer, epithelial ovarian carcinoma, melanoma, colon cancer, and brain tumors [6–9]. CD133 (prominin-1), a five-transmembrane glycoprotein, is commonly used as a surface marker for the identification of normal human stem cells. Previous studies showed that purified CD133^+^ cells generated neurospheres in culture and promoted brain tumors in in vivo models [6, 10–12]. In the present study, we aimed at isolating and culturing CSCs from rat C6 and human U87 tumor cell lines. CSCs were purified via selection with CD133 magnetic microbeads. We found that STAT3 inhibitor increased the sensitivity of glioma cells to chemotherapeutic drugs. Thus, concurrent targeting of CD133^+^ and CD133^−^ cells may be an effective therapeutic strategy for patients with malignant glioma.

## Methods

### Animals

Mice were housed in animal rooms with controlled temperature (23 ± 2 °C) and humidity (55 ± 5%), exposed to a 12-h light-dark cycle, and allowed free access to water and food. All experimental procedures were in accordance with the National Institutes of Health guidelines and were approved by the National Cheng Kung University Medical Center Animal Care and Use Committee (project approval number #104064).

### Cell culture

The human glioma cell line U87 was kindly provided by Dr. Michael Hsiao (Genomics Research Center, Academia Sinica, Taiwan) and rat glioma C6 cells was kindly provided by Dr. Shun-Fen Tzeng (National Cheng Kung University, Taiwan). The human glioma U87 cell line was cultured in Dulbecco’s modified Eagle medium (DMEM, Caisson) supplemented with 10% fetal bovine serum (FBS, Sigma-Aldrich), 2 mM L-glutamine (Caisson), 100 U/ml penicillin, and 0.1 mg/ml streptomycin (Caisson). The rat glioma C6 cell line was cultured in DMEM/F12 (Caisson) supplemented with 10% FBS, 2 mM L-glutamine, 100 U/ml penicillin, and 0.1 mg/ml streptomycin. Cultured cells were maintained in a humidified incubator at 37 °C in 5% CO_2_/95% air. The cells were labeled with 1 ml CD133/L micromagnetic beads per million cells using a CD133 cell isolation kit (Miltenyi Biotec, Bergisch Gladbach, Germany). CD133^+^ and CD133^−^ cells were plated onto 24-well culture dishes (5000 cells/well). CD133^+^ cells were plated in serum-free medium containing 10 μg/ml fibroblast growth factor 2 (FGF-2) and 10 μg/ml epidermal growth factor (EGF) and gave rise to non-adherent spheres on Ultra Low Attachment Multiple Well Plates (CORNING). CD133^+^ cells were allowed to form spheres/aggregates in a suspension culture, and were then dissociated and passaged using Accutase Cell Detachment Solution (BD Biosciences) at 37 °C for 30 min.

### Magnetic cell sorting and flow cytometry

C6 and U87 glioblastoma parental cells were trypsinized and suspended with ice-cold phosphate-buffered saline (PBS), centrifuged at 800 g for 5 min, and then resuspended in 1 × PBS with 0.5% bovine serum albumin (BSA) and 2 mM EDTA. Magnetically labeled anti-CD133 antibody from the Miltenyi Biotec CD133 cell isolation kit was used to isolate glioma CD133^+^ cells, as previously described [13]. CD133-PE conjugated antibody was applied for cell staining and evaluating the efficiency of magnetic separation via flow cytometry. The cell suspension was then placed within an autoMACS separator for magnetic separation. Labeled cells migrated toward the magnet; the unlabeled cells in suspension were drawn off. The remaining (labeled) cells were resuspended and then returned to the separator for further separation. The magnetic separation procedure was repeated twice to increase the efficiency of the magnetic separation. After the final elusion of the positive fraction of interest, the harvested cells suspended in culture medium were allowed for the downstream application. The separation purity was conducted via flow cytometry with a FACSCalibur machine (BD Biosciences).

### Cell viability assay

Glioma cells (2 × 10^3^ cells per well) were seeded in 96-well plates. Culture medium containing vehicle or drugs was added to the medium of each well, and cells were incubated at 37 °C for the indicated time. Cytotoxicity assayed via 2-(4-iodophenyl)-3-(4-nitrophenyl)-5-(2,4-disulfophenyl)-2H–tetrazolium monosodium salt (WST-1) reagent was used to measure cell viability. After aspirating drugs from wells, WST-1 was diluted in fresh culture medium (1:10) to a final volume of 100 μl and added into each well. The absorbance of soluble formazan was measured at 440 nm with a microplate reader. Cell viability is presented as the percentage of survivors relative to the vehicle-treated control culture. The absorbance of soluble formazan was measured at 440 nm with a microplate reader (Molecular Devices).

We used the response additivity approach [14]. In this approach, a positive drug combination effect occurs when the observed combination effect (*E*_*AB*_) is greater than the expected additive effect given by the sum of the individual effects (*E*_*A*_ + *E*_*B*_). The combination index (CI) was calculated as: CI = (E_A_ + E_B_)/E_(A + B)_.

### Western blotting assay

Glioma cells were treated with medium containing minocycline (Mino), WP1066, or vehicle in a 10-cm dish at 37 °C. At the indicated time, cells were centrifuged at 4000 rpm and pellets were collected and stored at −80 °C. Cell pellets were lysed in a lysis buffer containing 50 mM Tris–HCl, pH 7.4, 150 mM NaCl, 1% Nonidet P-40, 0.25% sodium deoxycholate, 0.1% sodium dodecyl sulfate (SDS), and supplemented with protease (Roche) and phosphatase inhibitors (Roche). Lysates were shaken at 40 rpm on ice for 1 h and then centrifuged at 13,000 rpm for 30 min at 4 °C. Supernatants were collected and protein concentration was measured via the Bradford assay. The proteins were re-suspended in a 5X sample buffer (12.5 mM Tris, 25% glycerol, 4% SDS, 1.54% DTT, and 0.02% Bromophenol blue) and boiled in water for 10 min. Protein electrophoresis was conducted on 15%, 10%, or 9% SDS-polyacrylamide gel under 100 V. The separated proteins were transferred to a PVDF membrane (Immunobilon transfer membranes, Millipore) using a semi-dry transfer system (BIO-RAD) under 400 mA and 20 V for 2 h. The membrane was then immersed in 5% nonfat milk or 3% BSA) for 1 h at room temperature for non-specific blocking; it was reacted at 4 °C overnight with the following primary antibodies: CD133 (1:6000, Merck Millipore), NANOG (1:1000, ProSci Inc.), SOX-2 (1:1000, abcam), Caspase 3 (1:2000, Cell Signaling), Caspase 8 (1:2000, Cell Signaling), Caspase 9 (1:2000, Cell Signaling), and β-Actin (1:400,000, Millipore). HRP-conjugated secondary antibody (Jackson ImmunoResearch Lab., USA) was incubated at room temperature for 1 h. After three rinses with TBST for 10 min each, ECL-plus chemical reagents (PerkinElmer) were added to the membrane. The films were exposed and developed until an optimal image was obtained, but not saturated. The films were scanned and images were analyzed and quantified using ImageJ software (NIH) to evaluate the expression of proteins of interest. The protein levels in all groups are expressed as a percentage of those in controls.

### Immunocytochemical staining of CD133^+^ cells

For immunostaining of non-adherent spheres, cells were seeded on Ultra Low Attachment Multiple Well Plates for 7 days. Cells were then fixed with 4% paraformaldehyde (PFA) in PBS and stained with primary antibodies against CD133 (mouse monoclonal; Merck Millipore), Nestin (rabbit polyclonal; Proteintech), SSEA-1 (rabbit polyclonal; Bioss), and SOX-2 (rabbit polyclonal; Abaca). The secondary antibodies used were Alexa Fluor®594-conjugated Goat-anti-rabbit IgG (Jackson ImmunoResearch) and Alexa Fluor®488-conjugated Sheep-anti-mouse IgG (Jackson ImmunoResearch). DAPI (4′,6-diamidino-2-phenylindole) (Sigma-Aldrich) dye was used to stain the nuclei.

### Neurosphere formation assay

To count the total number of neurospheres, CD133^+^ and CD133^−^ cells were suspended and seeded at 2.5 × 10^4^ cells/well in Ultra Low Attachment Multiple Well Plates in stem cell medium. After incubation for 14–21 days at 37 °C, floating neutrospheres were counted using inverted fluorescence microscopy (Olympus IX71). A cluster of more than five single cells was counted as a neurosphere.

### In vivo intracranial xenograft animal model and bioluminescence imaging

The lentiviral vector pAS2.EGFP construct was obtained from the National RNAi Core Facility at Academia Sinica, Taipei, Taiwan. Firefly luciferase cDNA was put into pAS2.EGFP and a bi-cistronic lentivirus expression vector was constructed. Lentiviruses were produced by co-transfecting the GFP-Luc-expressing lentiviral vector, the envelope plasmid (pMD2.G), and the packaging plasmid (pCMV-dR8.91) into 293 T cells using calcium phosphate. The culture medium was changed on day 2, and the viral supernatants were harvested and titrated.

For tumorigenesis, 10- to 12-week-old male nude mice (BALB/cAnN-Foxnlnu/CrlNarl mice, National Laboratory Animal Center) were subcutaneously injected with 1 × 10^4^ CD133^+^ or CD133^−^ cells. For the intracranial tumor model, U87 and C6 glioma cells were transduced with a lentiviral vector expressing GFP and firefly Luc. Luciferase-expressing CD133^+^ cells (5 × 10^3^ cells in Fig. 3b, 1 × 10^5^ cells in Fig. 7) were injected intracranially into the 10- to 12-week-old male nude mice. The sorted cells were immediately implanted into the nude mice without further culture/amplification. Nude mice were anesthetized with chlorohydrate and placed on a stereotaxic device. Subsequently, a Hamilton syringe with a 30-gauge needle was mounted on the stereotaxic device. U87 luciferase-expressing glioma cells were injected 1.5 mm caudal and lateral to the bregma, and at a depth of 3.5–4 mm into the left side of the brain. Ten days after tumor implantation, Mino (50 mg/kg in saline with 10% DMSO), WP1066 (20 mg/kg in DMSO and polyethylene glycol), or their combination was injected intraperitoneally once per day for 10 days into the mice. Tumors were monitored via longitudinal bioluminescence imaging, for which mice were injected with 100 μg of luciferin (Caliper), simultaneously anesthetized with isoflurane, and subsequently imaged with a cooled charge-coupled device camera (IVIS-200, Xenogen). Tumor light output was quantitated using the Living Image 2.5 software package (Xenogen).

**Fig. 1:**
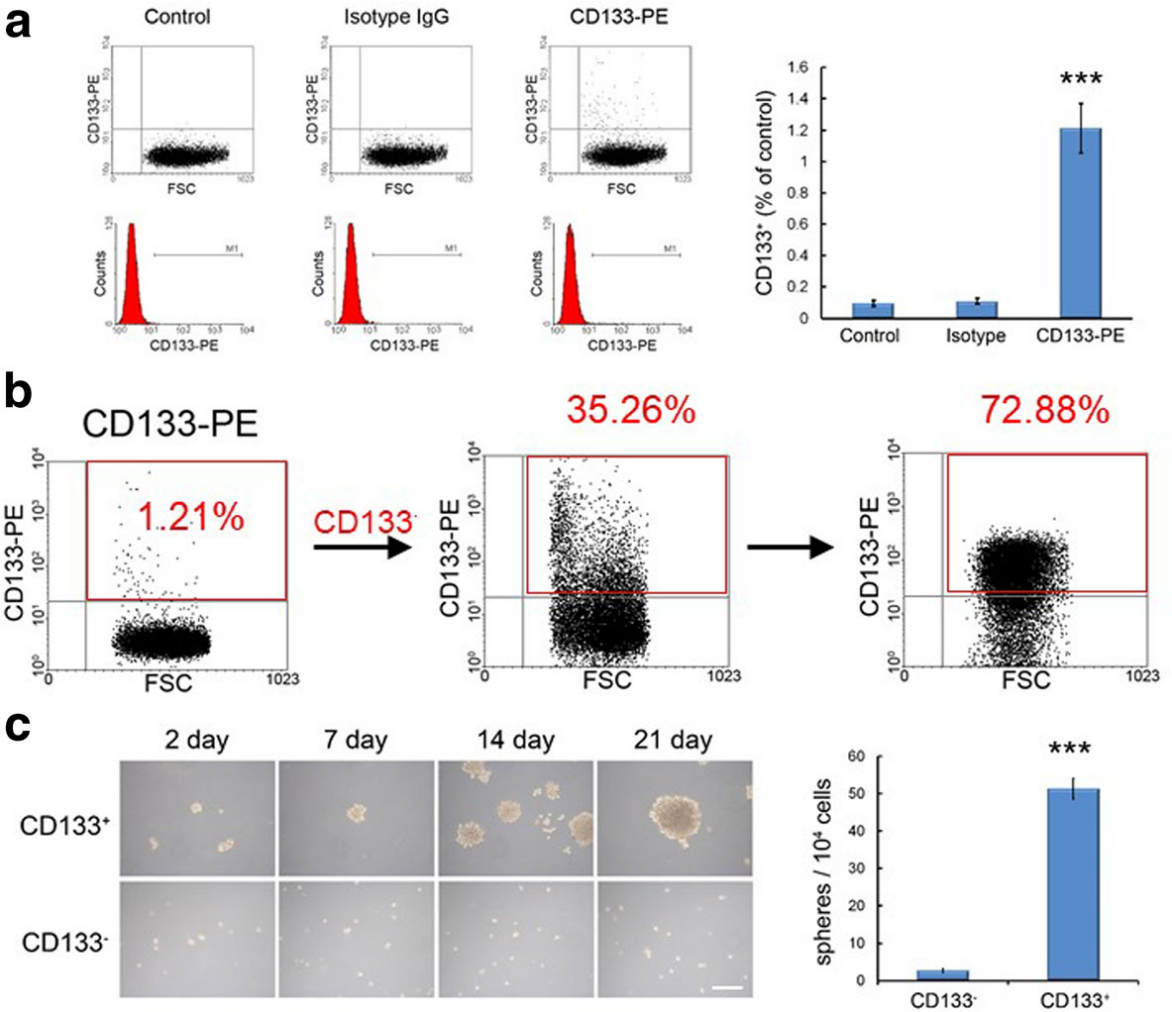
Analysis, sorting, and characterization of CD133 positive cells via flow cytometry in C6 glioma cells. **a** Flow cytometry analysis of CD133 expression in C6 glioma cells. We detected 1.21% CD133 positive cells in C6 glioma cells. ***p < 0.001 vs. control. **b** C6 glioma cells were sorted for CD133 expression using magnetic bead cell sorting. CD133^+^ cells were collected and cultured under the same conditions as those for unsorted parental cells. Purity of the CD133^+^ populations was 35.26% at the second sorting and 72.88% after the third sorting. **c** Primary neurospheres derived from CD133^+^ or CD133^−^ cells of C6 glioma cells were dissociated and cultured. CD133^+^ cells generated a greater number of secondary neurospheres than did CD133^−^ cell. ***p < 0.001 vs. CD133^−^. Scale bar: 100 μm

The method used to calculate combination drug interaction was based on previous reports [15, 16]. Fractional tumor volume (FTV) was calculated as tumor volume experimental/mean tumor volume control. Expected FTV was calculated as FTV of Minocycline × FTV of WP1066. A synergistic effect is suggested when the ratio of expected FTV/observed FTV is more than 1. A ratio of <1 indicates a less than additive effect.

### Statistical analysis

Experiments were performed at least in triplicate. All results are presented as mean ± standard error of the mean (SEM). Independent experiments were analyzed using the unpaired *t* test. One-way analysis of variance (ANOVA) was used to analyze differences in neurosphere numbers, various signaling inhibitors, and cell viability. Bonferroni multiple comparison tests were used as post hoc comparisons. Data were considered significant at the *p <* 0.05 level.

## Results

CD133 has been demonstrated as a marker of brain normal and tumor stem cells [6, 17]. Analyzing CD133 expression via flow cytometry revealed that 1.21% of C6 glioma cells were CD133 positive (CD133^+^) (Fig. 1a). Magnetic bead cell sorting was used to sort for CD133 expression in C6 glioma cells [18, 19]. Sorted CD133^+^ and CD133^−^ aliquots were checked with flow cytometry. The purity of the CD133^+^ populations was 35.26% at the second sorting and 72.88% after the third sorting. Only 0.48% of the CD133^−^ populations responded to CD133 antibody in the second sorting (Fig. 1b).

Primary neurospheres derived from CD133^+^ or CD133^−^ cells were dissociated and cultured. Over 3- to 21-day culture periods, CD133^+^ cells generated a greater number of secondary neurospheres than did CD133^−^ cells. CD133^+^ cells were capable of growing as non-adherent spheres and continued to expand their population. Unpaired *t* tests showed that the self-renewal ability of CD133^+^ cells at day 21 was significantly higher than that of CD133^−^ cells (t_(6)_ = 17.19, *p* < 0.001) (Fig. 1c). Similar isolation of CD133^+^ cells was performed from U87 glioma cells. A previous study revealed that the CD133^+^ cell fraction accounted for 0.5% of the total population in U87 cells [20]. The number of neurosheres derived from CD133^+^ cell at day 14 was significantly greater than that derived from CD133^−^ cells (*t*_(4)_ = 11.28, *p* < 0.001).

**Fig. 2:**
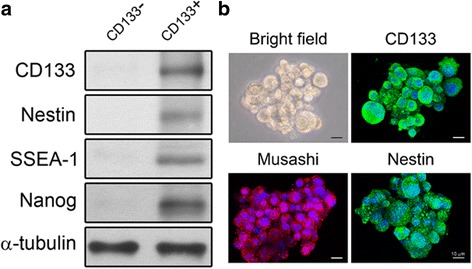
Neurospheres derived from CD133 positive cells exhibit stem cell-like markers. Western blotting (**a**) and immunochemical staining (**b**) of neurospheres derived from CD133^+^ cells. The neurospheres were positive for nestin, NANOG, and SSEA-1, markers for neural stem cells, embryonic stem cells, and pluripotent stem cells respectively. Scale bar: 10 μm

Nestin, a cytoskeletal protein, is known to be a neural stem/progenitor cell marker [21]. NANOG is a transcription factor important for the self-renewal of embryonic stem cells [22, 23]. Stage-specific embryonic antigen 1 (SSEA-1) is a marker of murine normal and stem-like cells [24]. Western blotting analysis showed that nestin, NANOG, and SSEA-1 were present in the CD133^+^ cells derived from C6 glioma cells (Fig. 2a). Furthermore, neurospheres derived from CD133^+^ cells were positive for nestin and Musashi, an RNA-binding protein that is selectively expressed in neural progenitor cells [25] (Fig. 2b). These stem cell markers were also present in the CD133^+^ cells derived from U87 glioma cells (data not shown).

**Fig. 3:**
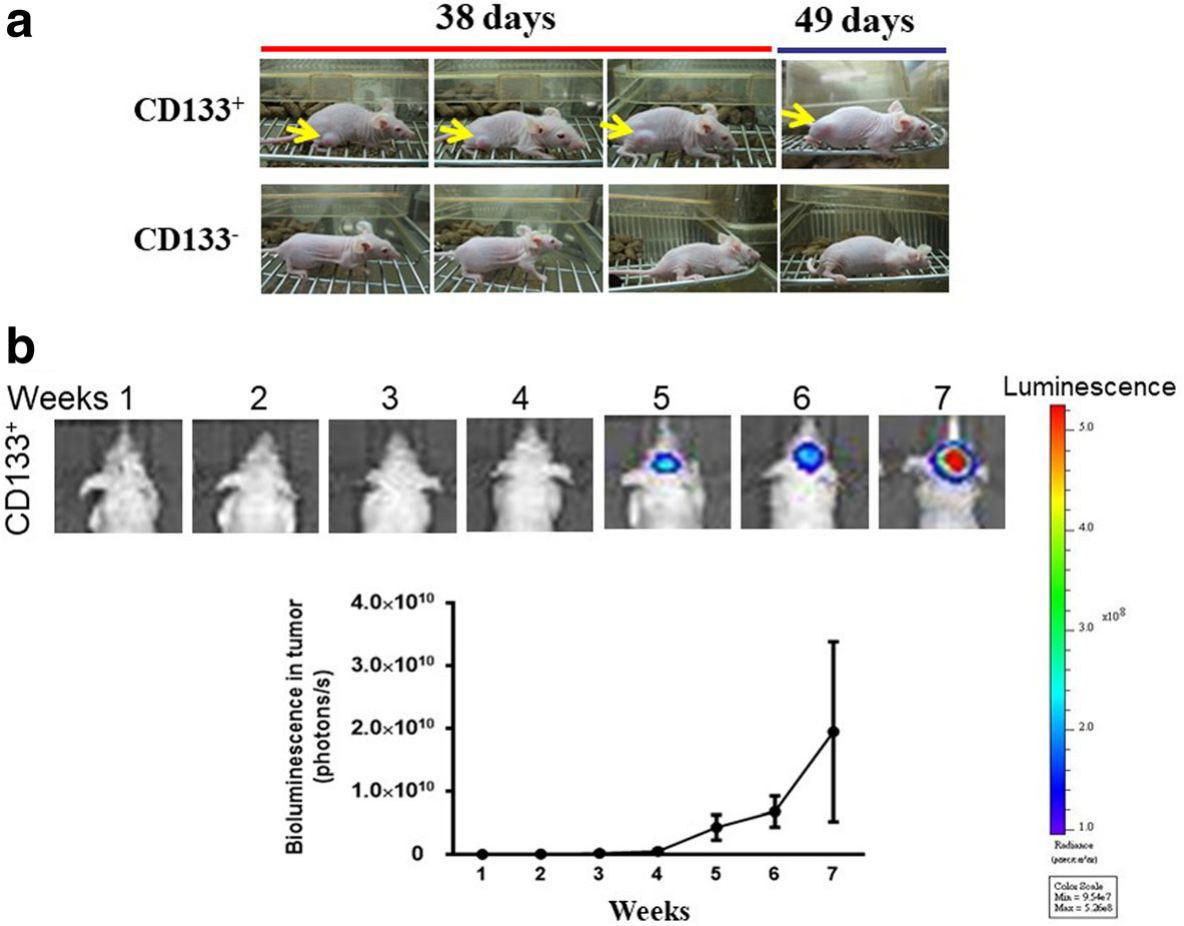
CD133^+^ but not CD133^−^ cells are able to form tumors in vivo. **a** CD133^+^ or CD133^−^ cells (1 × 10^4^) derived from C6 glioma cells were inoculated subcutaneously into the nude mice. Ten days after the inoculation, tumors were observed in 6 out of 6 mice inoculated. In contrast, no tumors formed in nude mice inoculated with CD133^−^ cells (0 out of 6 mice tested) (Fisher’s exact test, *p* < 0.01). **b** Luc-expressing CD133^+^ cells derived from U87 glioma cells were injected intracranially into athymic mice, and tumor growth was assessed using the IVIS-200 imaging system. Tumors were observed in 4 out of 4 mice injected intracranially with CD133^+^ cells. No tumors formed in 4 out of 4 mice injected with CD133^−^ cells (Fisher’s exact test, *p* < 0.05)

To address whether CD133^+^ and CD133^−^ cells differed in their ability to form tumors in vivo, we inoculated CD133^+^ or CD133^−^ cells derived from C6 glioma cells (1 × 10^4^) subcutaneously into the nude mice. Ten days after the inoculation, tumors were observed in 6 out of 6 mice inoculated with CD133^+^ cells. In nude mice inoculated with CD133^−^ cells, in contrast, no tumors formed (0 out of 6 mice tested) (Fisher’s exact test, *p* < 0.01) (Fig. 3a). We determined whether CD133^+^ cells promoted tumor formation in an intracranial tumor model. To monitor intracranial tumor growth, Luc-expressing CD133^+^ cells (5 × 10^3^ cells) derived from U87 glioma cells were injected intracranially into athymic mice, and tumor growth was assessed using the IVIS-200 imaging system. Consistently, tumors were observed in 4 out of 4 mice injected intracranially with CD133^+^ cells. No tumors formed in nude mice injected with CD133^−^ cells (0 out of 4 mice tested, Fisher’s exact test, *p* < 0.05) (Fig. 3b).

**Fig. 4:**
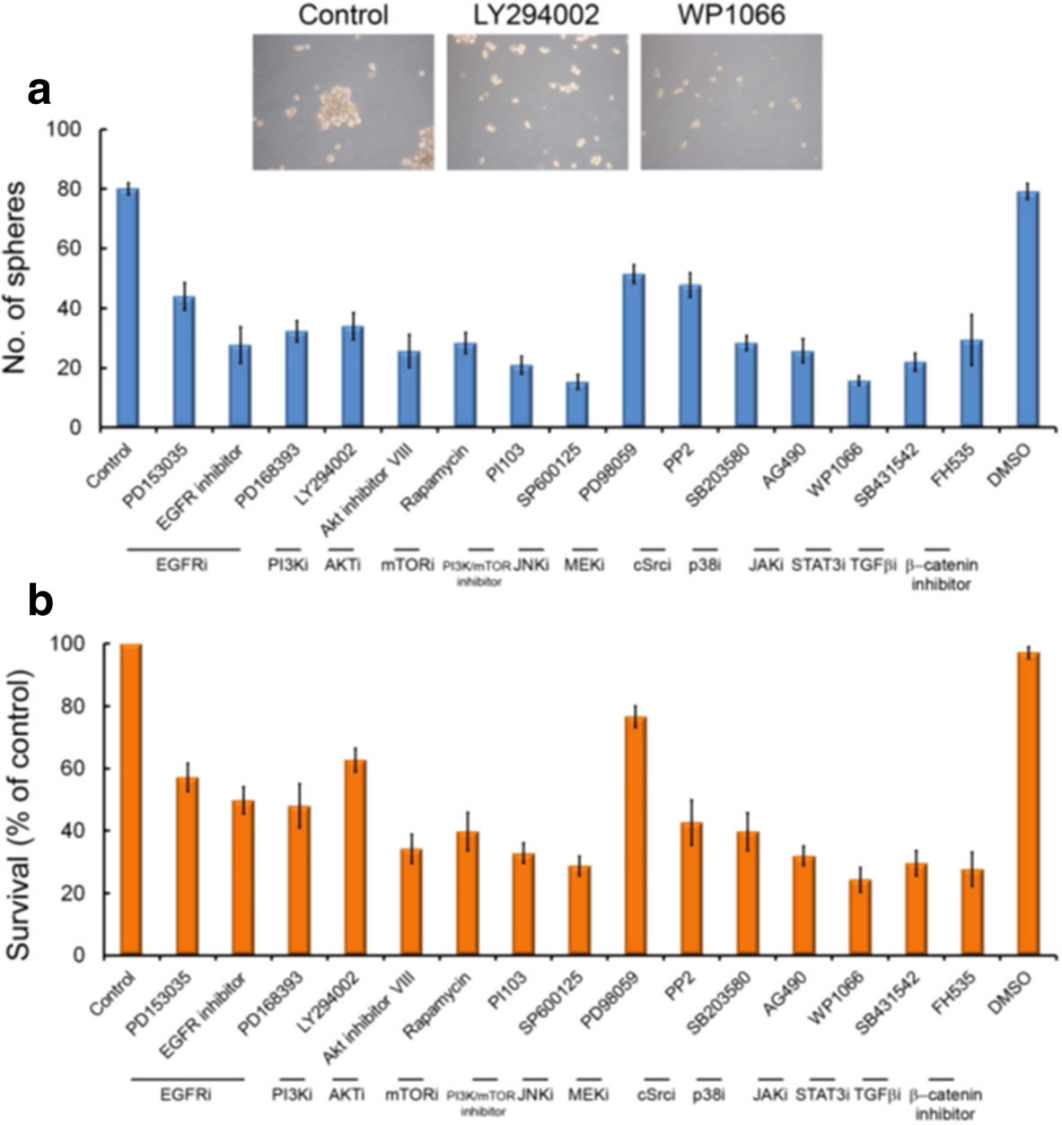
Effects of various signal pathway inhibitors on the number of neurospheres derived from CD133^+^ cells and the survival rate of C6 glioma cells. **a** Primary neurospheres derived from CD133^+^ were dissociated and cultured. They were then treated with EGFR inhibitors (PD103035 and PD168393), PI3K inhibitor (LY294002), Akt inhibitor (Akt inhibitor VIII), mTOR inhibitors (rapamycin, Pl103), JNK inhibitor (SP600125), MEK inhibitor (PD98059), cSrc inhibitor (PP2), p38 MEK inhibitor (SB203580), JAK inhibitor (AG490), STAT3 inhibitor (WP1006), TGFβ inhibitor (SB431542), or β-catenin inhibitor (FH535) for 24 h and number of neurospheres were measured. **b** CD133^+^ cells were treated with various signal pathway inhibitors for 48 h and the survival rate was measured using the WST-1 assay

We determined the signal pathways associated with neutrosphere formation activity by testing the effect of various signal pathway inhibitors on the self-renewal capacity of CD133^+^ cells derived from C6 glioma cells. CD133^+^ cells were treated with EGFR inhibitors (PD153035 and PD168393) [26, 27], PI3K inhibitor (LY294002) [28], Akt inhibitor (Akt inhibitor VIII) [29], mTOR inhibitors (rapamycin, Pl103), JNK inhibitor (SP600125), MEK inhibitor (PD98059), cSrc inhibitor (PP2) [30], p38 MEK inhibitor (SB203580), JAK inhibitor (AG490) [31], STAT3 inhibitor (WP1006) [32], TGFβ inhibitor (SB431542) [33], or β-catenin inhibitor (FH535) [34] for 24 h and the number of neurospheres was measured. As shown in Fig. 4a, STAT3 inhibitor exhibited a potent effect on reducing the number of neutrospheres derived from CD133^+^ cells. In parallel, CD133^+^ cells were treated with various signal pathway inhibitors for 24 h and the survival rate was determined using the WST-1 assay. STAT3 inhibitor also had a potent effect on reducing the survival rate of CD133^+^ cells (Fig. 4b).

**Fig. 5:**
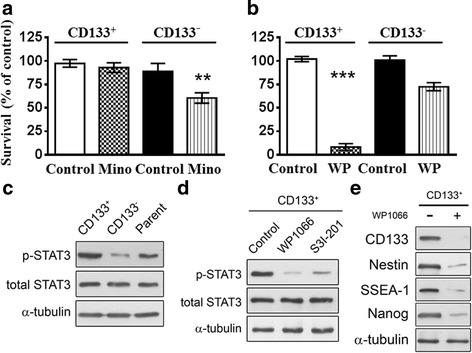
Effects of minocycline and STAT3 inhibitor on the survival rates of CD133^+^ and CD133^−^ cells from C6 glioma cells. (**a** and **b**) CD133^+^ and CD133^−^ cells were treated with Mino (25 μM) (**a**) or WP1066 (5 μM) (**b**) for 48 h and cell viability was assessed using the WST-1 assay. **p < 0.01, ***p < 0.001 CD133^+^vs. CD133^−^. **c** Western blotting analysis of p-STAT3 and STAT3 in CD133^+^, CD133^−^, and their parent cells from C6 glioma cells. **d** The phosphorylated states of STAT3 in CD133^+^ were markedly inhibited by STAT3 inhibitors WP1066 and S3 l-201. **e** Cancer stem cell markers Nestin, SSES-1, and NANOG in CD133^+^ were markedly inhibited by STAT3 inhibitor WP1066

Previously, we showed that Mino induced cell death in C6 glioma cells [35]. We compared the sensitivities of CD133^+^ and CD133^−^ cells to Mino treatment. CD133^+^ and CD133^−^ cells derived from U87 cells were treated with Mino (25 μM) for 48 h and cell viability was measured using the WST-1 assay. As shown in Fig. 5a, CD133^−^ cells were more sensitive to Mino than were CD133^+^ cells. Mino at a concentration of 25 μM decreased the number of CD133^−^ cells by 40% but only decreased the number of CD133^+^ cells by 7% (t_(8)_ = 4.271, *p* < 0.01). Conversely, STAT3 inhibitor WP1066 at a concentration of 5 μM reduced the number of CD133^+^ cells by 92% but only reduced the number of CD133^−^ cells by 27.5% (t_(18)_ = 11.29, *p* < 0.001) (Fig. 5b).

We also compared the activated states of STAT3 among CD133^+^, CD133^−^, and their parental cells from C6 glioma cells and found that CD133^+^ exhibited the highest phosphorylated state (Fig. 5c). CD133^+^ C6 glioma cells were treated with STAT3 inhibitors WP1066 (5 μM) or S3 l-201 (50 μM) [36] for 24 h and p-STAT3, STAT3, CD133, Nestin, SSES-1, and NANOG were measured using Western blotting analysis. As expected, the phosphorylated states of STAT3 were markedly inhibited by WP1066 and S3 l-201 (Fig. 5d), so as the cancer stem cell markers Nestin, SSES-1, and NANOG (Fig. 5e).

**Fig. 6:**
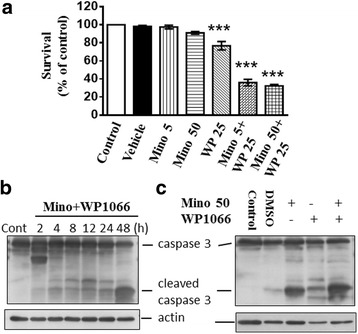
Effects of Mino and WP1066 alone or in combination on the cell survival and caspase-3 activation in U87 glioma cells. **a** U87 glioma cells were treated with Mino (5 or 50 μM) and WP1066 (25 μM) alone or in combination and cell survival was assessed 48 h after the treatment using the WST-1 assay. Mino plus WP1066 synergistically inhibited cell growth. ***p < 0.001 vs. control. **b** Western blotting analysis of cleaved caspase-3. U87 glioma cells were treated with Mino (50 μM) and WP1066 (5 μM) for the indicated times. The expression of the cleaved fragment of caspase 3 was significantly increased. **c** U87 glioma cells were treated with Mino (50 μM), WP1066 (5 μM), or their combination for 48 h. The expression of the cleaved fragment of caspase 3 was determined using Western blotting analysis

We determined whether Mino (5 μM) alone or in combination with WP1006 induced synergistic cytotoxicity toward glioma cells. U87 glioma cells were treated with Mino (5 μM), WP1006 (25 μM), or Mino (5 μM) plus WP1006 (25 μM) and cell viability was assessed using the WST-1 assay. Mino alone reduced survival rate by 2.6% whereas WP1006 reduced survival rate by 23.3%. Mino plus WP1006 inhibited cell growth by 64% (Fig. 6a). The combination drug index (CDI) for WP1006 (25 μM) and Mino (5 μM) was 0.481. Similarly, Mino (50 μM) alone reduced survival rate by 9% and Mino plus WP1006 inhibited cell growth by 67.7%. CDI for WP1006 (25 μM) and Mino (50 μM) was 0.462. The CDI values are less than 1, indicating a synergistic effect.

U87 glioma cells were treated with Mino (50 μM) plus WP1066 (5 μM) for different times, as indicated. We found that the cleaved fragment of caspase 3 was increased at 48 h after treatment (Fig. 6b). Furthermore, U87 glioma cells were treated with Mino (50 μM), WP1066 (5 μM), or their combination for 48 h. The expression of the cleaved fragment of caspase 3 was determined using Western blotting analysis. Figure 6c shows that the expression of the cleaved fragment of caspase 3 was higher after a combined application of Mino (50 μM) and WP1006 (5 μM) than those after the application of Mino or WP1006 alone.

**Fig. 7:**
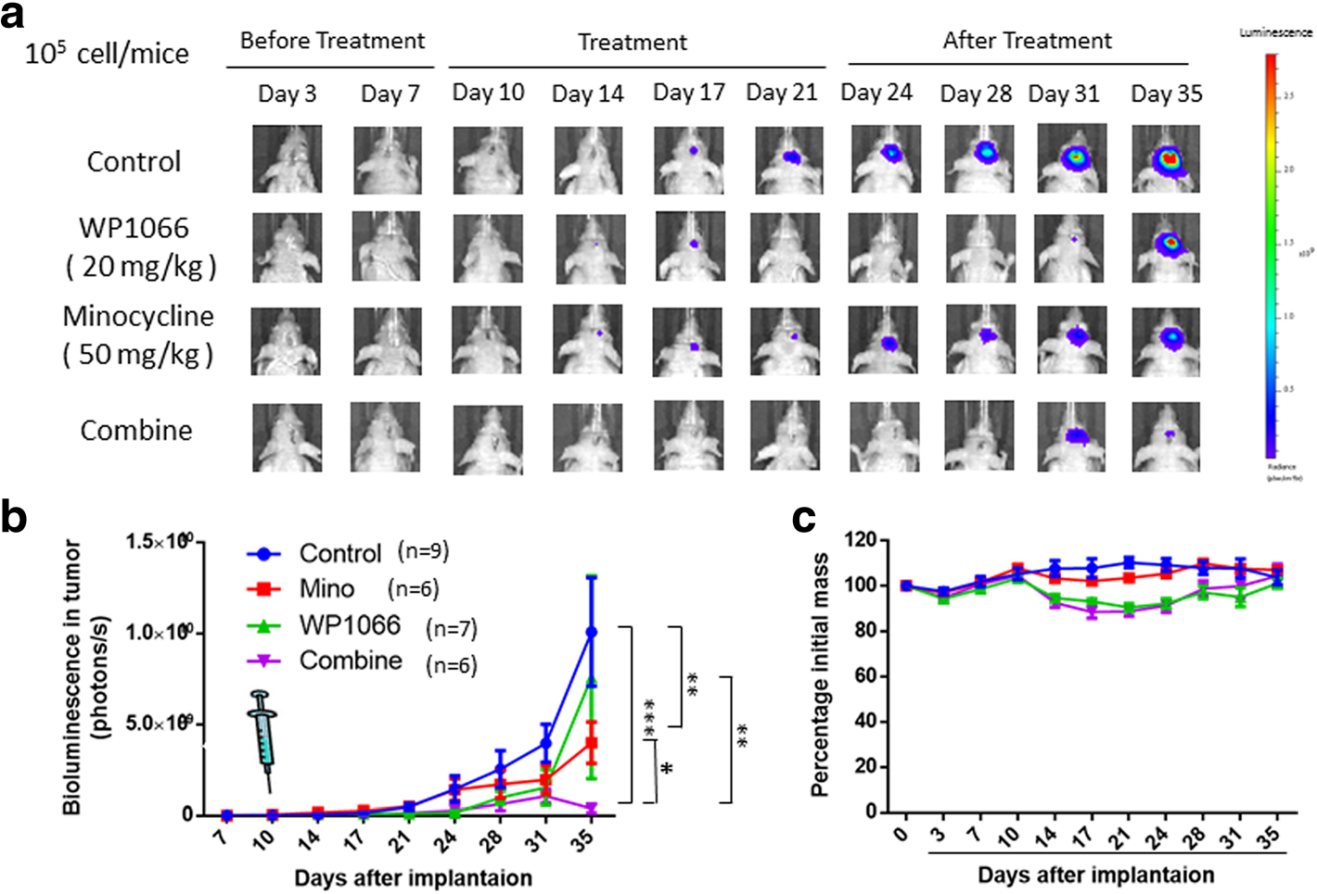
Minocycline and WP1066 in combination synergistically inhibit intracranial tumor growth. **a** U87 glioma cells were injected intracranially into athymic mice and tumor growth was studied using the IVIS-200 imaging system. **b** At day 10 after intracranial injection of tumor cells, Mino (50 mg/kg), WP1006 (20 mg/kg), or their combination was administered intraperitoneally once per day for 10 days and tumor growth was observed for 15 days after the cessation of treatment. **c** Weight measurements were taken every 4 days. There were no differences among the four groups at day 35

We used the intracranial tumor model to determine whether Mino plus WP1006 could synergistically inhibit tumor growth. Transduced glioma cells were injected intracranially into athymic mice (Fig. 7a). At day 10 after injection, Mino (50 mg/kg), WP1006 (20 mg/kg), or their combination was injected intraperitoneally once per day into the mice for 10 days and tumor growth was observed for 15 more days (Fig. 7b). At day 35, Mino inhibited tumor growth by 63.3% and WP1006 inhibited tumor growth by 13%. Mino plus WP1006 in combination inhibited tumor growth by 98.9%. The expected fractional tumor volume (FTV) was 0.319 and the observed FTV was 0.011. The ratio of expected FTV/observed FTV was 29. A ratio of >1 indicates a synergistic effect and a ratio of <1 indicates a less than additive effect [15, 16]. These results suggest that a combination of Mino and WP1006 synergistically inhibits the intracranial growth of U87 glioma cells. The body weight of the mice was measured every 4 days. There were no differences among control, Mino, WP1006, and combination groups at day 35 (Fig. 7c).

## Discussion

Although brain tumors are composed of a heterogeneous mass of cells, a subpopulation of cells called tumor stem cells is capable of self-renewal and initiating the formation of neurospheres [3–5]. Here, we established a method for the isolation, culture, and purification of tumor stem cells from rat C6 and human U87 glioblastoma cell lines using magnetic beads coupled to anti-CD133 antibody. These CD133^+^ cells exhibited immunophenotypic characteristics of neural stem cells. First, CD133^+^ cells were capable of initiating the formation of neurospheres, whereas CD133^−^ cells were unable to form tumors at the cell number tested. Second, neurospheres derived from CD133^+^ cells were positive for nestin, NANOG, and SSEA-1, markers for neural stem cells, embryonic stem cells, and pluripotent stem cells, respectively. Third, when CD133^+^ cells were inoculated into the nude mice, they were able to form tumors in vivo. In contrast, CD133^−^ cells were unable to form tumors. These results indicate that we successfully established a process for the isolation and culture of glioblastoma stem cells.

In the present study, we demonstrated that the survival of CD133^+^ stem-like cells in glioblastoma depends on STAT3 activity. We showed that the phosphorylated and activated level of STAT3 was higher in CD133^+^ cells than in CD133^−^ cells. STAT3 inhibitor WP1066 exhibited a potent effect on decreasing the number of neurospheres derived from CD133^+^ cells. In addition, the survival rate of glioma cells and the expression of cancer stem cell markers Nestin, SSES-1, and NANOG were attenuated by WP1066. These results are consistent with previous reports showing that the STAT3 signaling pathway contributes to the progression of neurosphere-initiating tumor cells [32, 33].

We next examined the effect of WP1066 in combination with Mino, which was more effective in reducing the survival of CD133^−^ cells than CD133^+^ cells. Mino plus WP1006 synergistically inhibited the survival of glioma cells in vitro as well as the intracranial growth of U87 glioma cells in vivo. Furthermore, the expression of the cleaved fragment of caspase 3 was increased, suggesting that the combination of Mino and WP1066 induced cell death through caspase-dependent apoptosis.

We found that inhibiting both CD133^+^ and CD133^−^ cells is more effective than inhibiting CD133^+^ cells only. The results suggest that CD133^−^ cells if untreated may undergo de-differentiation or reprogramming such that they can be converted to CD133^+^ cells. Since STAT3 inhibitor reduced the viability of CD133^+^ cells, STAT3 activation may promote reprogramming. Previous reports showing that STAT3 signaling is sufficient to promote somatic cell reprogramming [37–39] support this hypothesis.

Chemotherapy has not effectively increased the survival of patients with GBM because it usually targets the fast growing tumor mass, leaving cancer stem cells less affected. Combination therapy, on the other hand, is advantageous because it may not only lower the nonspecific toxicity produced by a high dose of single treatment but can also target different subpopulations of cancer cells. There may be adverse effects, as several inhibitors have been withdrawn from clinical trials due to serious side effects, including systemic STAT3 inhibition [40, 41].

## Conclusion

We isolated cancer stem-like cells from human U87 and rat C6 glioblastoma cells via magnetic cell sorting using CD133 as a marker. We found that Mino was more effective in reducing the viability of CD133^−^ cells, whereas STAT3 inhibitor was more effective in reducing the viability of CD133^+^ cells. Mino and STAT3 inhibitor in combination produced a synergistic effect in reducing the cell viability of glioma cells in vitro and inhibited tumor growth in nude mice. This suggests that simultaneously targeting different subpopulations of glioblastoma cells may be an effective therapeutic strategy for patients with malignant glioma.

## Abbreviations

CDI: Combination drug index
CSCs: Cancer stem cells
FTV: Fractional tumor volume
GBM: Glioblastoma Multiforme
GSCs: Glioma stem cells
Mino: Minocycline
STAT3: Signal transducer and activator of transcription 3
TICs: Tumor-initiating cells
WST-1: 2-(4-iodophenyl)-3-(4-nitrophenyl)-5-(2,4-disulfophenyl)-2H–tetrazolium
